# Critical and distinct roles of cell type–specific NF-**κ**B2 in lung cancer

**DOI:** 10.1172/jci.insight.164188

**Published:** 2024-02-22

**Authors:** Fan Sun, Yadong Xiao, Steven D. Shapiro, Zhaoxia Qu, Gutian Xiao

**Affiliations:** 1 UPMC Hillman Cancer Center, Department of Microbiology and Molecular Genetics, University of Pittsburgh School of Medicine, Pittsburgh, Pennsylvania, USA.; 2 Norris Comprehensive Cancer Center, Hastings Center for Pulmonary Research, Department of Molecular Microbiology and Immunology, University of Southern California Keck School of Medicine, Los Angeles, California, USA.; 3 Division of Pulmonary, Allergy, and Critical Care Medicine, Department of Medicine, University of Pittsburgh School of Medicine, Pittsburgh, Pennsylvania, USA.; 4 Department of Medicine, University of Southern California Keck School of Medicine, Los Angeles, California, USA.

**Keywords:** Oncology, Cancer, Lung cancer

## Abstract

Different from the well-studied canonical NF-κB member RelA, the role of the noncanonical NF-κB member NF-κB2 in solid tumors, and lung cancer in particular, is poorly understood. Here we report that in contrast to the tumor-promoting role of RelA, NF-κB2 intrinsic to lung epithelial and tumor cells had no marked effect on lung tumorigenesis and progression. On the other hand, NF-κB2 limited dendritic cell number and activation in the lung but protected lung macrophages and drove them to promote lung cancer through controlling activation of noncanonical and canonical NF-κB, respectively. NF-κB2 was also required for B cell maintenance and T cell activation. The antitumor activity of lymphocyte NF-κB2 was dominated by the protumor function of myeloid NF-κB2; thus, NF-κB2 has an overall tumor-promoting activity. These studies reveal a cell type–dependent role for NF-κB2 in lung cancer and help understand the complexity of NF-κB action and lung cancer pathogenesis for better design of NF-κB–targeted therapy against this deadliest cancer.

## Abstract 2

<p>NF-&kappa;B2 function in lung cancer pathogenesis is cell-type specific, as nonimmune cell-derived NF-&kappa;B2 is trivial and myeloid-derived NF-&kappa;B2 drives protumor activity that previals over anti-tumor activity of lymphocyte-derived NF-&kappa;B2.</p>

## Graphical abstract

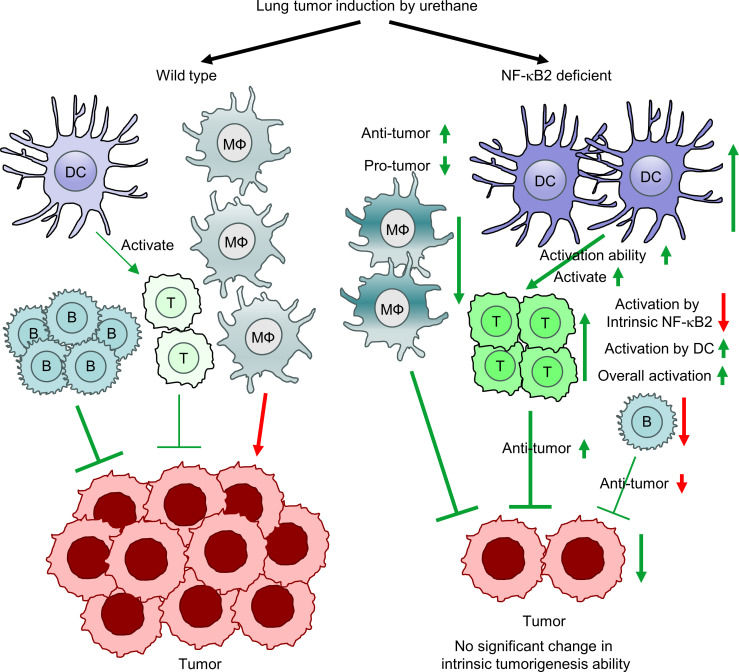

## Introduction

The master transcription factor NF-κB has been linked to almost all human cancers and in particular lung cancer, the second most common cancer type and the leading cause of cancer-related deaths, with a 5-year survival rate of only 22% and annual deaths of over 130,000 Americans (1–3). Aberrant NF-κB activation has been suggested to be involved in the full process of tumor development, from initiation to metastasis, as well as cancer therapy resistance (1, 2). Mechanistically, NF-κB contributes to tumor pathogenesis, both intrinsically and extrinsically. Within precancerous or cancerous cells, activated NF-κB regulates a wide range of genes not only to promote malignant cell survival, proliferation, invasion, metastasis, and immune evasion, but also to induce angiogenesis and tumorigenic inflammation (1, 2). Moreover, NF-κB activated in tumor-associated cells, particularly immune cells, contributes to tumor pathogenesis indirectly through establishing a protumorigenic microenvironment (4, 5). Accordingly, it is not surprising that NF-κB and in particular its key activator, IKK kinase, has been a target of great interest for tumor therapy during the past several decades (6). However, we have been unable to successfully target it for therapy due to its functional complexities (6, 7).

NF-κB is not a single protein, but refers to 5 structurally related transcription factors: NF-κB1, NF-κB2, RelA (also known as p65), RelB, and c-Rel (1, 2). The tumorigenic roles of NF-κB in lung and other cancers are largely derived from the antitumor effects of NF-κB inhibition by administration of IKK inhibitors, overexpression of the NF-κB inhibitor IκBα, and the knockout/knockdown of IKK or RelA (1, 2, 8–14). But, both IKK and IκBα have many NF-κB–independent activities that are also implicated in tumorigenesis (15, 16). Furthermore, they control the activation of NF-κB members other than RelA (1, 2). Different NF-κB members may also have different or even contrasting functions, although they usually form and function as dimers (1, 2). In this regard, RelA activation in lung tumor cells or tumor-associated myeloid cells/macrophages (TAMs) is associated with disease progression and poor patient survival (5, 10, 12, 14). Genetic deletion of RelA in lung precancerous and cancerous cells or TAMs substantially, although incompletely, blocks lung tumorigenesis in mouse models of lung cancer (5, 12, 14). In contrast, deletion of NF-κB1 from mice increases lung cancer induction, and NF-κB1 expression is positively associated with lung cancer patient survival (17). Intriguingly, the lung tumor suppressive function of NF-κB1 is independent of its NF-κB functions: its precursor form p105 as an NF-κB inhibitor or its mature form p50 as a functional NF-κB member (17). Instead, it depends on p105 stabilization of the kinase Tpl2 (also known as Cot), which in turn prevents lung damage and inflammation and oncogenic mutations (17). Of note, RelA and p50 usually function as the heterodimer that is often simply referred to as NF-κB, the canonical NF-κB (1).

To better understand the complexity of NF-κB action and lung cancer pathogenesis for better design of NF-κB–targeted therapy against this deadliest cancer, it is thus of importance and interest to determine the roles of NF-κB2 as well. Like NF-κB1, NF-κB2 protein exists as 2 forms, p100 and p52, the precursor and mature forms, respectively (18). p100 serves as an inhibitor of NF-κB by sequestering NF-κB members and in particular RelA and RelB in the cytoplasm, whereas p52 dimerizes with RelB as an important and functional NF-κB that is known as the noncanonical NF-κB (18).

To define the role of NF-κB2 in lung cancer, we exploited NF-κB2–null mice (*Nfkb2*^–/–^ mice, referred to as NF-κB2–KO mice hereafter) for the impact of NF-κB2 deficiency on lung tumorigenesis induced by ethyl carbamate (also called urethane). Urethane is a chemical carcinogen present in fermented food, alcoholic beverages, and cigarette smoke that accounts for approximately 90% of lung cancer cases in humans (19–21). Urethane-induced lung cancers in mice faithfully recapitulate their human counterparts and in particular adenocarcinomas associated with tobacco smoking, the most common type of lung cancer that makes up approximately 40% of all lung cancers, and therefore has been widely used to study the mechanisms underlying lung tumorigenesis (22–25).

## Results

### NF-κB2–deficient mice are much more resistant to lung cancer.

**Figure 1:**
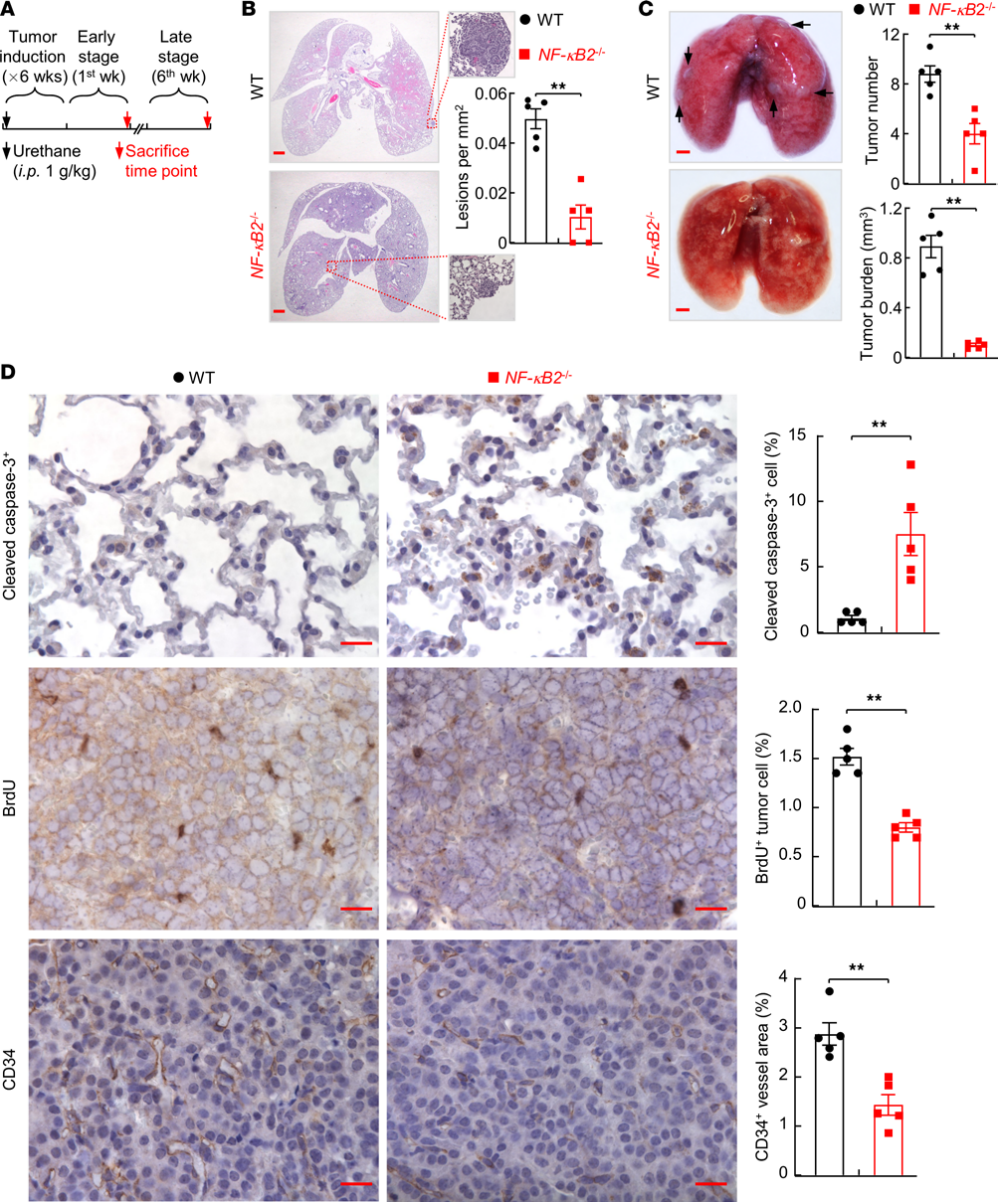
Decreased lung cancer induction in NF-κB2–deficient mice. (**A**) Schedule of lung cancer induction and mouse analysis. (**B**) Histological analysis showing decreased lung lesions in NF-κB2–deficient mice 1 week after urethane treatment. Scale bar: 1 mm. (**C**) Lung examination showing decreased lung tumor number and burden in NF-κB2–deficient mice 6 weeks after urethane treatment. Scale bars: 1 mm. (**D**) IHC staining of lung sections showing increased tumor cell apoptosis but decreased tumor cell proliferation and tumor angiogenesis in urethane-treated NF-κB2–deficient mice. Cleaved caspase-3–, BrdU-, and CD34-positive cells were counted and are represented as a percentage of total cells. Scale bars: 20 μm. Data are presented as mean ± SEM (*n* = 5). **P < 0.01 by 2-tailed, unpaired Student’s *t* test (**B**–**D**).

NF-κB2–KO mice are healthy and fertile and show normal lung development and function, at least under pathogen- and treatment-free conditions (refs. 26, 27; also see Figure 1, B and C). After exposure to urethane, all wild-type (WT) or NF-κB2–KO mice developed lung tumors (Figure 1, A–C). However, NF-κB2–KO mice had markedly fewer lung lesions at early stages and significantly fewer tumors at late stages of lung tumorigenesis. Moreover, the tumors in NF-κB2–KO mice were significantly smaller than those in WT mice. Consistent with this observation, cleaved caspase 3, BrdU, and CD34 staining of tumor tissues indicated that lung tumors in NF-κB2–KO mice had increased cell death, reduced cell proliferation, and decreased angiogenesis, in comparison with those in WT mice (Figure 1D). These data suggested that NF-κB2 promotes lung tumor initiation and progression.

### The decreased lung cancer in NF-κB2–deficient mice is mainly caused by NF-κB2 deficiency in immune cells but not in nonimmune cells or lung cancer cells in particular.

**Figure 2:**
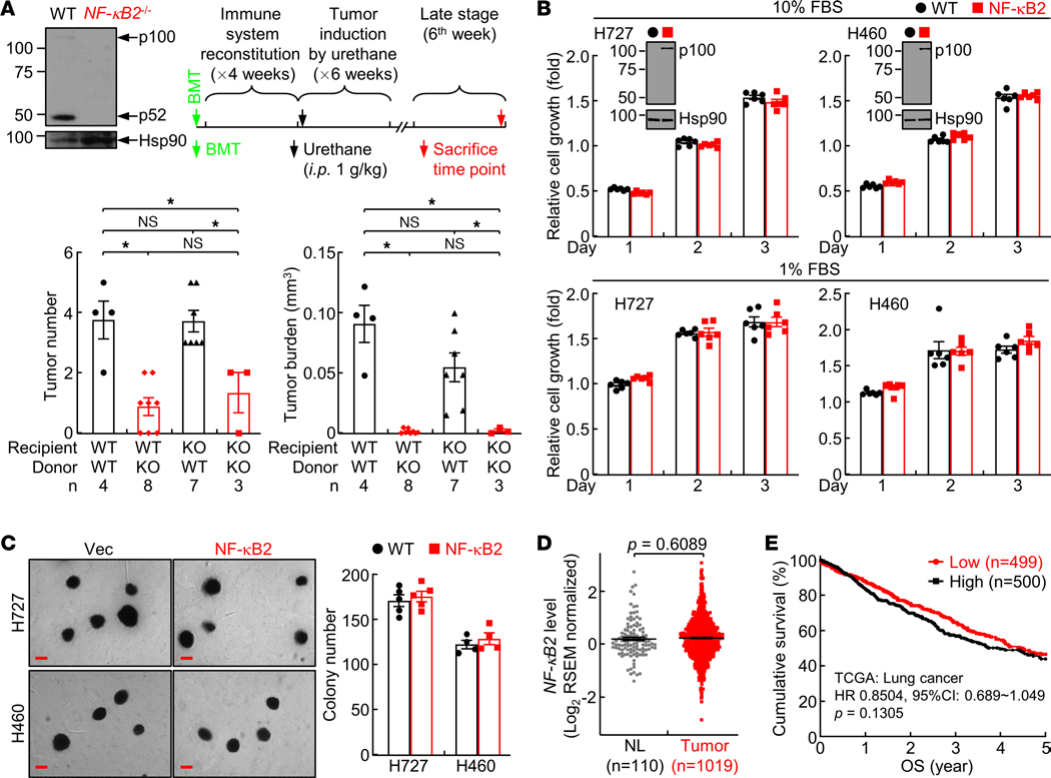
Lung tumor suppression by immune, but not nonimmune, NF-κB2. (**A**) Bone marrow transplantation assays showing lung tumor suppression by immune, but not nonimmune, NF-κB2. NF-κB2 KO was confirmed by IB analysis of the spleen from the donor mice. Sample number (*n*) is indicated at the bottom for each group. (**B**) Cell growth assays showing no effect of ectopic NF-κB2 on human lung cancer cell growth in culture (*n* = 6). Ectopic NF-κB2 (p100) expression was confirmed by IB analysis. (**C**) Soft agar colony formation assays showing no effect of ectopic NF-κB2 on anchorage-independent growth of H727 (*n* = 5) and H460 (*n* = 4) human lung cancer cells. Scale bars: 200 μm. (**D**) TCGA data showing no change in NF-κB2 expression in human lung cancers. (**E**) Kaplan-Meier survival analysis showing no association of NF-κB2 expression in lung tumors with patient survival. Data are presented as mean ± SEM. *P < 0.05 by ordinary 1-way ANOVA (**A**), 2-tailed, unpaired Student’s *t* test (**B**–**D**), or log-rank test (**E**). NS, not statistically significant.

To determine whether the decreased lung tumorigenesis in NF-κB2–KO mice is caused by NF-κB2 deficiency in immune cells and/or nonimmune cells, and in particular lung epithelial and tumor cells, we generated NF-κB2–KO or WT bone marrow–chimeric (BM-chimeric) mice for the in vivo lung tumorigenesis assays (Figure 2A). As evidenced by fewer and smaller lung tumors, lung tumorigenesis induced by urethane was significantly decreased in NF-κB2–KO mice that received NF-κB2–KO BM cells, compared with WT mice that received WT BM cells. This copied the difference between NF-κB2–KO and WT mice that did not receive BM transplantation. Notably, similar lung tumor suppression was found in WT mice that received NF-κB2–KO BM cells, but the lung tumor suppression was blocked in NF-κB2–KO mice that received WT BM cells. These data indicated that NF-κB2 intrinsic to immune cells, but not to nonimmune cells, is required for lung cancer promotion.

To validate the dispensable role of NF-κB2 intrinsic to nonimmune cells, and in particular lung tumor cells, we examined the effect of ectopic NF-κB2 on the tumorigenicity of human lung cancer cells in vitro. Stable expression of NF-κB2 did not affect the growth of the human lung cancer cell lines H727 and H460 in culture medium containing 10% or 1% fetal bovine serum (FBS) (Figure 2B). It also did not affect their anchorage-independent cell growth, as indicated by no effect on their colony-forming ability in soft agar (Figure 2C). Analysis of The Cancer Genome Atlas (TCGA) data indicated that NF-κB2 expression was not changed in human lung tumors compared to normal lung tissues (Figure 2D). Moreover, NF-κB2 expression in tumors was not associated, either positively or negatively, with patient survival (Figure 2E). These data together suggested that NF-κB2 intrinsic to immune cells promotes lung tumorigenesis, whereas NF-κB2 in nonimmune cells, including lung tumor cells, does not contribute much to lung cancer pathogenesis.

### B cells and their intrinsic NF-κB2 are required for lung cancer suppression.

**Figure 3:**
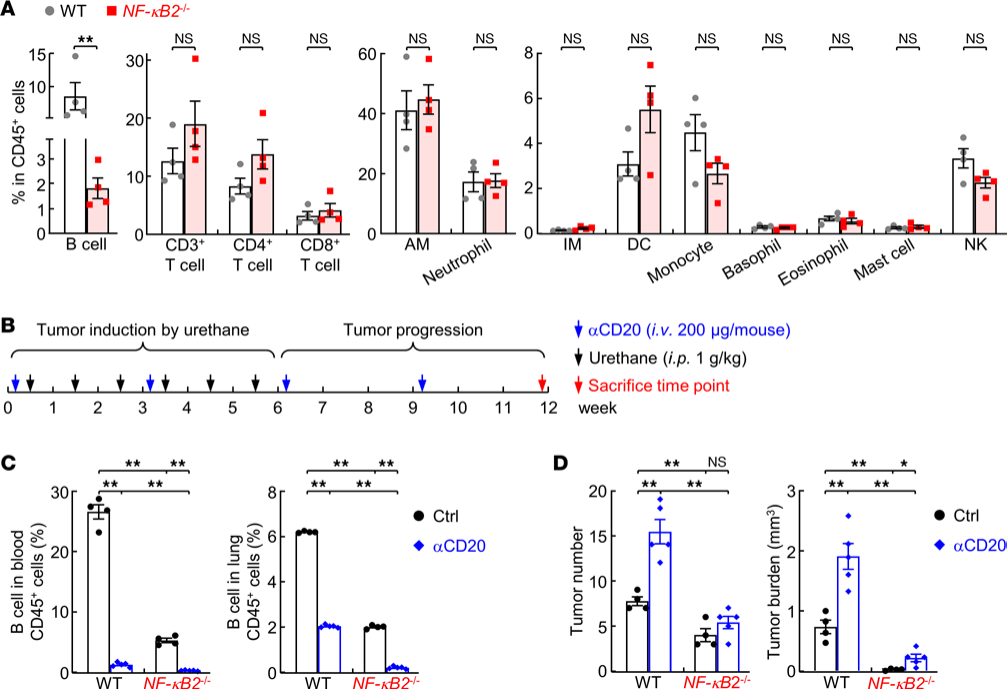
Requirement for B cells and their intrinsic NF-κB2 for lung cancer suppression. (**A**) Flow cytometry analysis showing decreased B cells but no changes in other immune cells in the lung of untreated NF-κB2–deficient mice (*n* = 4). AM, alveolar macrophage; IM, lung interstitial macrophage; DC, dendritic cell; NK, natural killer cell. (**B**) Schedule of B cell depletion by anti-CD20. (**C**) Flow cytometry analysis showing efficient B cell depletion in mouse blood and lung. Ctrl, *n* = 4; anti-CD20, *n* = 5. (**D**) B cell depletion assays showing the requirement for B cells and their intrinsic NF-κB2 for lung cancer suppression. Ctrl, *n* = 4; anti-CD20, *n* = 5. Data are presented as mean ± SEM. *P < 0.05; **P < 0.01 by 2-tailed, unpaired Student’s *t* test (**A**) or ordinary 1-way ANOVA (**C** and **D**). NS, not statistically significant.

To define the immune cell type(s) in which NF-κB2 is required to promote lung cancer, we analyzed the pulmonary immune profiles of NF-κB2–KO mice and WT mice. NF-κB2 deficiency led to an over 80% reduction in pulmonary B cells, but had no significant effect on other immune cells in the lung under pathogen- and treatment-free conditions (Figure 3A). Nevertheless, this is highly in agreement with the cell-autonomous role of NF-κB2 in B cell survival and maintenance during B cell development (18, 26).

To determine whether the decrease in B cells is the mechanism underlying the decreased lung cancer in NF-κB2–KO mice, B cells were depleted from WT mice with anti-CD20 antibodies starting before lung cancer induction (Figure 3B). As a control, NF-κB2–KO mice were included in parallel. As shown in Figure 3C, B cells were efficiently depleted in mouse bloods and lungs. However, B cell depletion resulted in much more and larger lung tumors in WT mice (Figure 3D). It also significantly increased lung tumor size in NF-κB2–KO mice. The lung tumor number in NF-κB2–KO mice was increased as well, though with no statistical significance. These data suggested a lung tumor–suppressive role for B cells and their intrinsic NF-κB2. They also suggested that the increased protumor activity in NF-κB2–KO mice caused by B cell NF-κB2 deficiency and B cell reduction is dominated by the increased antitumor immunity induced by NF-κB2 deficiency in immune cells other than B cells, resulting in the overall decreased lung cancer in NF-κB2–KO mice.

### Cell-intrinsic NF-κB2 contributes to T cell antitumor activity.

**Figure 4:**
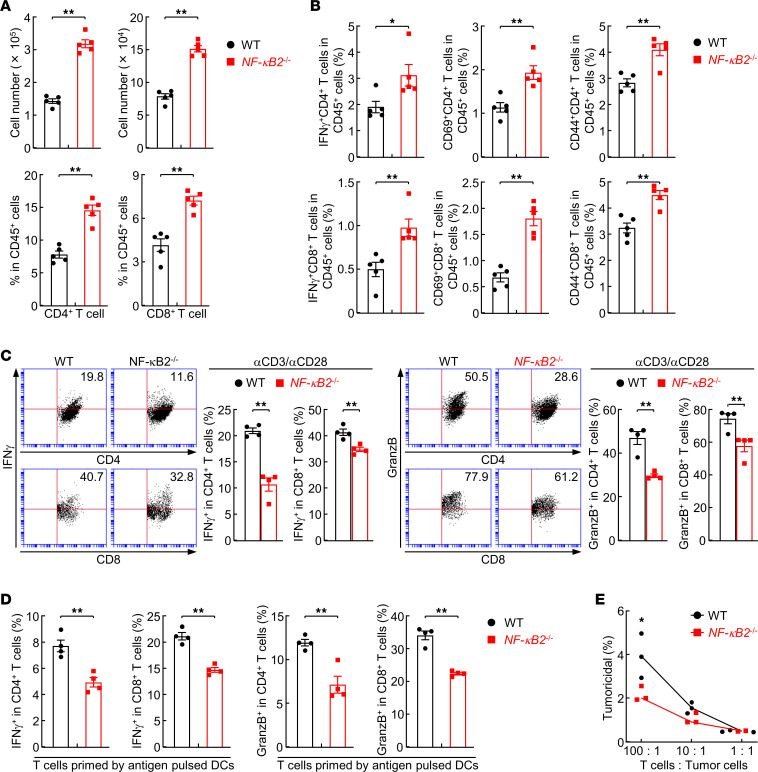
Indispensable role of intrinsic NF-κB2 in T cell antitumor activity. (**A**) Flow cytometry analysis showing increased T cells in the lung of urethane-treated NF-κB2–deficient mice (*n* = 5). (**B**) Flow cytometry analysis showing more activated T cells in the lung of NF-κB2–deficient mice treated with urethane (*n* = 5). (**C**) Flow cytometry analysis showing decreased activation of NF-κB2–deficient T cells induced by anti-CD3 and anti-CD28 antibodies (*n* = 4). (**D**) Flow cytometry analysis showing impaired activation of NF-κB2–deficient T cells by tumor antigen–loaded DCs (*n* = 4). (**E**) In vitro tumor cell killing assays showing impaired tumoricidal activity of NF-κB2–deficient T cells activated by tumor antigen-loaded DCs (*n* = 3). Data are presented as mean ± SEM. *P < 0.05; **P < 0.01 by 2-tailed, unpaired Student’s *t* test (**A**–**E**).

Although there was no significant difference in untreated mice, the numbers of CD4^+^ and CD8^+^ T cells were markedly increased in the lung of NF-κB2–KO mice 6 weeks after urethane treatment (Figure 4A). Moreover, their antitumor activity was significantly higher, as evidenced by the significant increase in cells expressing the activation markers interferon γ (IFN-γ), CD69, and CD44 (Figure 4B). The increase in these adaptive immune cells correlated with the decreased lung tumorigenesis in NF-κB2–KO mice.

To determine whether the increased antitumor T cells are induced directly by the deletion of intrinsic NF-κB2 and/or indirectly by its deletion in other immune cells such as dendritic cells (DCs), we compared in vitro the activation of T cells purified from the spleen of untreated NF-κB2–KO and WT mice by using anti-CD3 plus anti-CD28. In contrast to our expectation, NF-κB2–KO T cells showed defective activation compared with WT T cells (Figure 4C). To validate and extend the studies, we performed in vitro assays of lung tumor antigen–dependent T cell activation and tumoricidal activity (28, 29). DCs derived from BM (BMDCs) of untreated WT mice were pulsed with lysates of the mouse lung tumor cell line LLC stably expressing luciferase (LLC-Luc), and then cocultured with NF-κB2–KO or WT T cells. NF-κB2–KO T cells exhibited a significantly lower activation by the pulsed DCs (Figure 4D). Accordingly, NF-κB2–KO T cells activated by DCs exhibited decreased cytotoxicity toward the LLC-Luc cells (Figure 4E). These data suggested that NF-κB2 actually serves as an intrinsic driver of T cells to suppress lung cancer. They also suggested that the reduced tumoricidal ability of NF-κB2–deficient T cells has been overcome in NF-κB2–KO mice, externally by NF-κB2 deficiency in immune cells other than T cells and B cells, for lung cancer suppression.

### NF-κB2 is an intrinsic inhibitor of DCs that blocks T cell antitumor activation.

**Figure 5:**
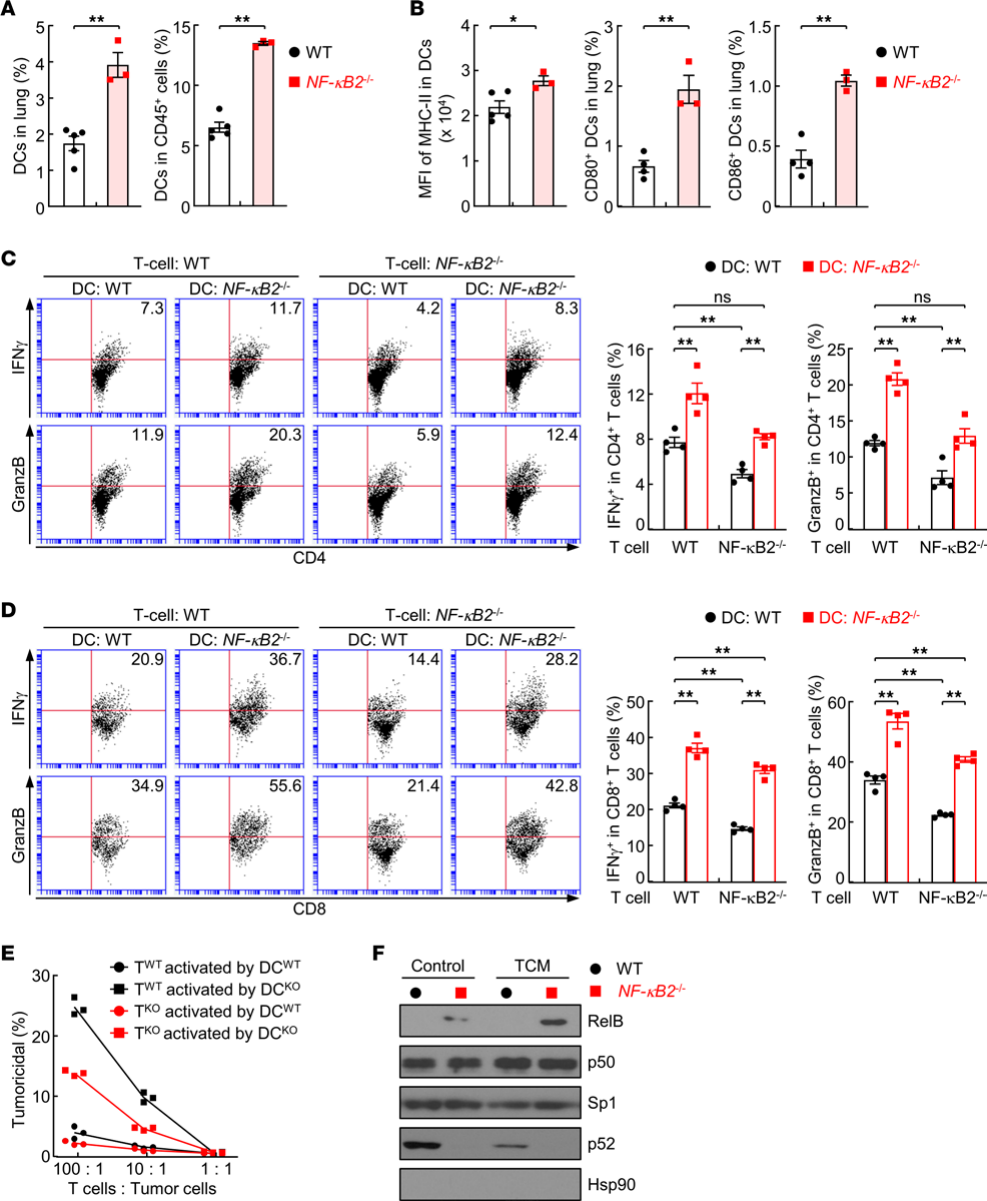
Increased T cell activation activity of DCs induced by NF-κB2 deletion in lung tumorigenesis. (**A**) Flow cytometry analysis showing increased DCs in the lung of urethane-treated NF-κB2–deficient mice (*n* = 3) compared with WT mice (*n* = 5). (**B**) Flow cytometry analysis showing increased MHC-II (WT, *n* = 5; *NF-κB2^–/–^*, *n* = 3), CD80 (WT, *n* = 4; *NF-κB2^–/–^*, *n* = 3), and CD86 (WT, *n* = 4; *NF-κB2^–/–^*, *n* = 3) in DCs, markers of DC activation, in the lung of urethane-treated NF-κB2–deficient mice. (**C**) In vitro T cell activation analysis showing increased ability of NF-κB2–deficient DCs in activating CD4^+^ T cells (*n* = 4). (**D**) In vitro T cell activation showing increased ability of NF-κB2–deficient DCs in activating CD8^+^ T cells (*n* = 4). (**E**) In vitro tumor cell killing assays showing increased ability of NF-κB2–deficient DCs in inducing the tumoricidal activity of T cells (*n* = 3). (**F**) IB of nuclear fraction showing increased RelB activation in NF-κB2–deficient DCs induced by TCM. Data are presented as mean ± SEM. *P < 0.05; **P < 0.01 by 2-tailed, unpaired Student’s *t* test (**A**, **B**, and **E**) or ordinary 1-way ANOVA (**C** and **D**). NS, not statistically significant.

Given the potent role of DCs in T cell activation against tumor cells, we hypothesized that NF-κB2 deficiency in DCs renders antigen-presenting cells (APCs) better able to activate T cells for lung cancer suppression. Indeed, significantly more DCs were found in the lung of the NF-κB2–KO mice during lung tumorigenesis induced by urethane (Figure 5A). Furthermore, pulmonary DCs in NF-κB2–KO mice expressed significantly higher levels of the antigen presentation molecule major histocompatibility complex class II (MHC-II) on the cell surface, and significantly more pulmonary DCs in NF-κB2–KO mice expressed the T cell costimulatory molecules CD80 and CD86 (Figure 5B).

To examine whether the increased pulmonary DCs and their activity contribute to the increased T cell activation and tumoricidal ability for lung cancer suppression in NF-κB2–KO mice, we compared in vitro the abilities of NF-κB2–KO and WT DCs in activating naive NF-κB2–KO or WT T cells. WT or NF-κB2–KO BMDCs were pulsed with LLC-Luc cell lysates and then cocultured with purified splenic T cells of untreated NF-κB2–KO or WT mice. Compared with coculture with WT DCs, indeed, coculture with NF-κB2–KO DCs resulted in drastically higher activity of both CD4^+^ and CD8^+^ T cells, regardless of their NF-κB2 expression status (Figure 5, C and D). In further support of our previous finding showing a positive role of cell-intrinsic NF-κB2 in T cell activation, the activation of NF-κB2–KO CD4^+^ or CD8^+^ T cells by either WT or NF-κB2–KO DCs was always lower than that of their WT counterparts. Similar results were obtained for their tumoricidal activity (Figure 5E and Supplemental Table 1; supplemental material available online with this article; https://doi.org/10.1172/jci.insight.164188DS1).

Consistent with the fact that NF-κB2 p100 is the main inhibitor of RelB, a key regulator of DCs (30–32), NF-κB2–KO DCs showed higher nuclear expression of RelB, a hallmark of RelB activation (Figure 5F), which was further increased by adding conditioned medium from the LLC-Luc lung tumor cells (TCM). These data suggested that NF-κB2 restricts DCs from activating T cells and thereby facilitates lung tumorigenesis.

### NF-κB2 acts as an intrinsic molecular guardian that also controls activation of lung macrophages for lung tumor promotion.

**Figure 6:**
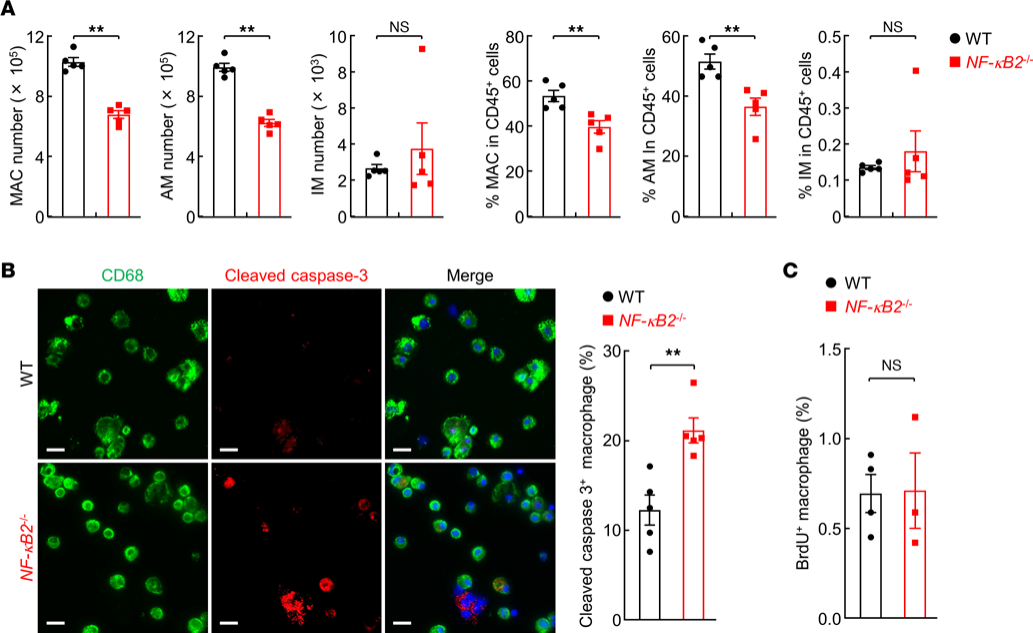
Increased apoptosis of NF-κB2–deficient macrophages during lung tumorigenesis. (**A**) Flow cytometry analysis showing decreased lung macrophages in urethane-treated NF-κB2–deficient mice (*n* = 5). (**B**) IF staining of cleaved caspase-3 showing increased AM apoptosis in urethane-treated NF-κB2–deficient mice (*n* = 5). Scale bars: 20 μm. (**C**) BrdU labeling showing no significant difference in the proliferation rate of AMs in urethane-treated NF-κB2–deficient (*n* = 3) or WT (*n* = 4) mice. Data are presented as mean ± SEM. **P < 0.01 by 2-tailed, unpaired Student’s *t* test (**A**–**C**). NS, not statistically significant.

Macrophages are the most abundant immune cells in the lung and within the tumor microenvironment that dictate the immune response under both physiological and pathogenic conditions (33–38). It is of interest and importance to define whether and how NF-κB2 regulates these key immune cells in lung tumorigenesis, which remains unknown. Despite comparable numbers in untreated mice, lung macrophages and alveolar macrophages (AMs) in particular were significantly decreased in NF-κB2–KO mice compared with WT mice after urethane treatment (Figure 6A). Lung interstitial macrophages (IMs) showed no such change. AMs are the most abundant immune cells within the lung and often simply referred to as lung macrophages. In line with the function of NF-κB2 p52 in the expression of cell survival genes and in particular its essential role in B cell survival and maintenance during B cell development (18, 26), significantly increased apoptosis of lung macrophages was detected in the urethane-treated NF-κB2–KO mice, in comparison with WT mice with the same urethane treatment (Figure 6B). On the other hand, the proliferation rates of those cells were similar (Figure 6C). These data suggested that NF-κB2 p52 is required for lung macrophage survival and maintenance during lung tumorigenesis.

**Figure 7:**
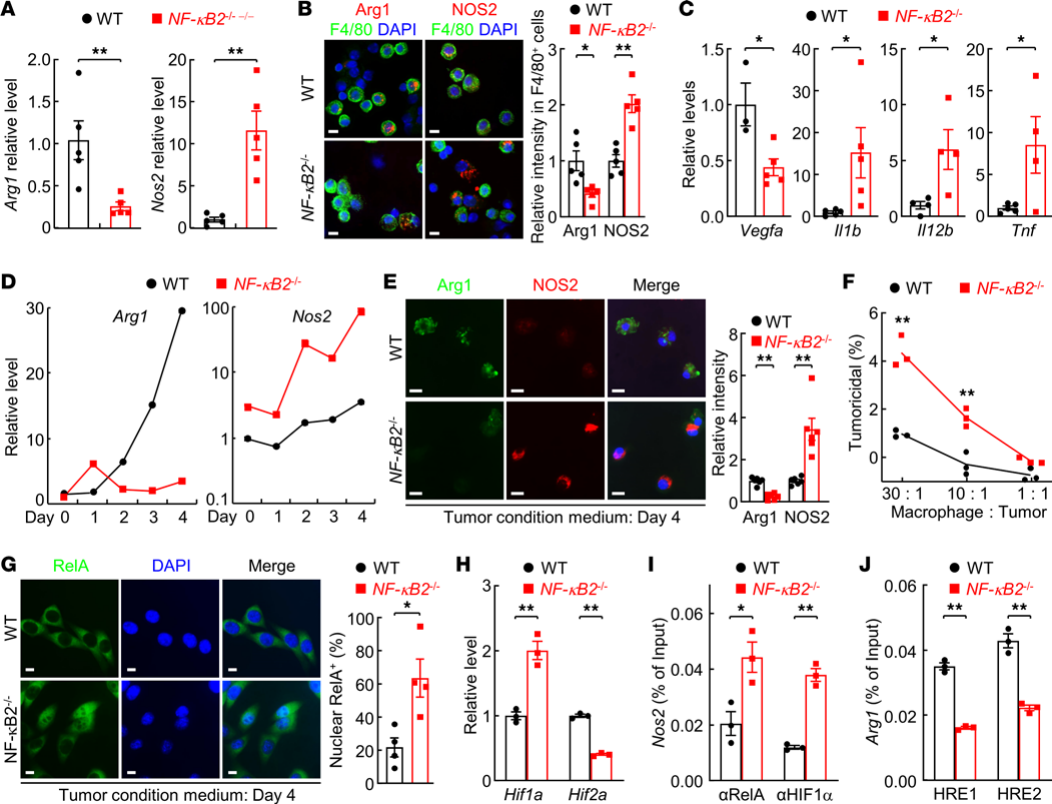
NF-κB2 regulation of AMs involving RelA inhibition. (**A**) qPCR showing decreased *Arg1* and increased *Nos2* in the AMs of urethane-treated NF-κB2–deficient mice (*n* = 5). (**B**) IF analysis showing decreased Arg1 and increased NOS2 in the AMs of urethane-treated NF-κB2–deficient mice (*n* = 5). (**C**) qPCR showing decreased *Vegfa* (WT, *n* = 3; *NF-κB2^–/–^*, *n* = 5), but increased *Il1b* (*n* = 5), *Il12b* (*n* = 4), and *Tnf* (WT, *n* = 5; *NF-κB2^–/–^*, *n* = 4) in the AMs of urethane-treated NF-κB2–deficient mice. (**D**) qPCR showing different increases in *Nos2* and *Arg1* in NF-κB2–deficient and WT macrophages by lung tumor–conditioned medium (TCM), respectively. (**E**) IF analysis showing increased NOS2 and decreased Arg1 proteins in NF-κB2–deficient (*n* = 6) compared with WT (*n* = 6) macrophages cultured with TCM. (**F**) In vitro tumor cell killing assays showing enhanced tumor-killing ability of NF-κB2–deficient macrophages activated by TCM (*n* = 3). (**G**) IF analysis showing higher nuclear RelA in NF-κB2–deficient macrophages cultured with TCM (*n* = 4). (**H**) qPCR showing increased *Hif1a* and decreased *Hif2a* in NF-κB2–deficient macrophages induced by TCM culture (*n* = 3). (**I**) ChIP assays showing increased RelA and HIF1α at the *Nos2* promoter in NF-κB2–deficient macrophages induced by TCM culture (*n* = 3). (**J**) ChIP assays showing decreased HIF2α binding to 2 hypoxia-response element (HRE) sites within the *Arg1* promoter in NF-κB2–deficient macrophages induced by TCM culture (*n* = 3). Scale bars: 10 μm (**B**, **E**, and **G**). Data are presented as mean ± SEM. *P < 0.05; **P < 0.01 by 2-tailed, unpaired Student’s *t* test (**A**, **C**, **F**, and **H**–**J**). NS, not statistically significant.

AMs in urethane-treated NF-κB2–KO mice also expressed significantly less arginase-1 (Arg1) but more inducible nitric oxide synthase 2 (NOS2) at both RNA and protein levels, in comparison with those in WT mice with the same urethane treatment (Figure 7, A and B). Arg1 is often used as a marker of protumor activation, while NOS2 is a well-established marker of macrophage antitumor responses (36). Consistently, AMs in urethane-treated NF-κB2–KO mice showed decreased expression of the tumor-promoting factor VEGFα, but increased expression of antitumor cytokines IL-1β, IL-12β, and TNF-α (Figure 7C).

To define the mechanism by which NF-κB2 deletion leads to increased antitumor activation but decreased protumor activation of lung macrophages, we first cultured in vitro the NF-κB2–KO and WT macrophages derived from the BM (BMDMs) of untreated mice with the LLC-Luc TCM. Despite the same basal levels in WT and NF-κB2–KO macrophages, *Arg1* mRNA was robustly induced by TCM in WT but not NF-κB2–KO macrophages (Figure 7D). On the other hand, the basal level of *Nos2* mRNA was already higher in NF-κB2–KO macrophages. This difference in *Nos2* mRNA expression became greater after TCM treatment, although TCM induced NOS2 in both NF-κB2–KO and WT macrophages. Consistently, more Arg1 protein in WT macrophages and more NOS2 protein in NF-κB2–KO macrophages was detected (Figure 7E). Compared with WT macrophages, NF-κB2–KO macrophages also showed significantly higher tumoricidal activity (Figure 7F). These studies suggested that the reduced cell number, the decreased protumor activation, and the increased antitumor activation of lung macrophages contribute to the decreased lung tumorigenesis in NF-κB2–KO mice. They also suggested that the in vitro system faithfully resembled the activation of lung macrophages of the NF-κB2–KO and WT mice during lung tumorigenesis.

Thus, we exploited the in vitro system for further mechanistic studies. In line with the increased expression of TNF-α and IL-1β (the prototypical activators of the canonical NF-κB) by NF-κB2–KO AMs, significantly more RelA protein was detected in the nuclei of NF-κB2–KO macrophages cultured in TCM (Figure 7G). Consistent with the fact that hypoxia-inducible factor-1α (HIF1α) is a transcription target of RelA (39, 40), higher HIF1α was detected in NF-κB2–KO macrophages (Figure 7H). Interestingly, a lower level of HIF2α was found in NF-κB2–KO macrophages. Accordingly, there was more RelA and HIF1α protein at the *Nos2* promoter, and less HIF2α protein at hypoxia-response element (HRE) sites within the *Arg1* promoter in NF-κB2–KO macrophages (Figure 7, I and J). Given the role of RelA and HIF1α in NOS2 induction and the role of HIF2α in Arg1 expression (41–43), these results suggested that loss of NF-κB2 results in RelA hyperactivation and HIF1α induction but HIF2α repression, leading to strong induction of NOS2 but not Arg1 in AMs and subsequently lung tumor suppression.

## Discussion

NF-κB2 p100 has 2 prototypical functions, as an inhibitor of NF-κB (IκB) by binding to and sequestering NF-κB members in the cytoplasm and as the precursor of NF-κB2 p52, a mature and functional member of NF-κB. In response to several stimuli and in particular those involved in B cell development, the C-terminal IκB-containing part of p100 is degraded by the proteasome, and the remaining N-terminal polypeptide (p52) enters the nucleus to regulate transcription of genes vital for B cell survival (18). NF-κB2 often undergoes genetic mutations in human blood tumors, accounting for approximately 1%–5% of human leukemia and lymphomas (18, 44, 45). The oncogenic mutations always lead to the generation of C-terminal partially truncated p100 mutants, which constitutively translocate into the nucleus and are processed at the NF-κB–containing promoters to become p52 for gene regulation and tumorigenesis (46). The tightly regulated processing of p100 for p52 generation is also aberrantly activated, independently of its genetic mutation, in certain cancers, such as multiple myeloma, adult T cell leukemia/lymphoma (ATL) by the oncogenic virus human T cell leukemia virus type 1 (HTLV-1), as well as Kaposi sarcoma and several other lymphoproliferative disorders induced by Kaposi’s sarcoma herpesvirus/human herpesvirus-8 (KSHV/HHV-8) (18, 47, 48). However, NF-κB2 mutations have rarely been found in solid tumors, and lung cancer in particular. In fact, whether and how NF-κB2 is involved in lung cancer has not been investigated until the studies above showed its important but complicated roles in lung tumorigenesis.

In contrast to the reported tumorigenic roles of NF-κB2, which depend on uncontrolled p100 processing to generate p52 as an oncogenic driver, intrinsic NF-κB2, either p100 or p52, is not required for lung tumor formation or maintenance. Genetic deletion of NF-κB2 in nonimmune cells, including lung epithelial, precancerous, and cancerous cells, has no marked effect on lung cancer pathogenesis. On the other hand, overexpression of NF-κB2 in lung cancer cells has no effect on their tumorigenicity either. Consistently, NF-κB2 expression is not changed in human lung cancers or associated with patient survival. This is also different from the tumor-promoting and -suppressive roles of intrinsic RelA and NF-κB1 in lung cancer, respectively (5, 12, 14). Therefore, these 3 NF-κB members in the cells of tumor origin and tumor cells play 3 different roles. It will be interesting to see wether this is also applicable to other cancer types. It would also be interesting to test the role of RelB and c-Rel in lung cancer, particularly given the fact that p100 is the main inhibitor and p52 is the main functional partner of RelB (18, 44).

**Figure 8:**
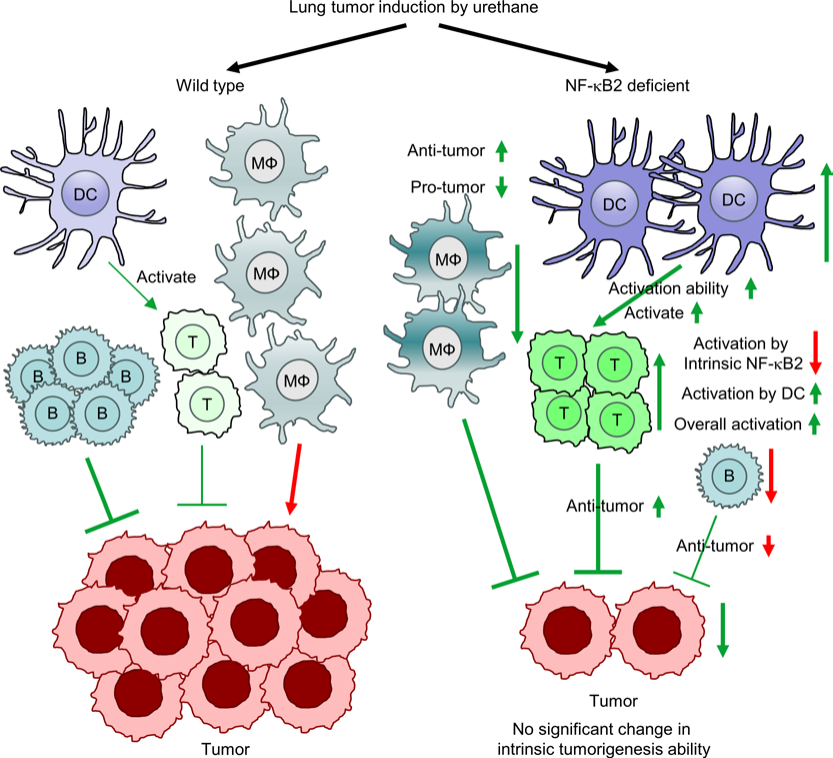
Schematic showing distinct roles for NF-κB2 in different cell types in lung cancer.

It is clear that NF-κB2 promotes lung cancer indirectly through governing immune cells. However, in different immune cell types, NF-κB2 plays different or even opposite roles, despite a net outcome of tumor promotion. In general, it is required for myeloid cells to promote, but for lymphocytes to suppress, lung tumors, and the tumor promotion function in myeloid AMs and DCs is dominant. In lung tumorigenesis, cell-intrinsic NF-κB2 enhances the protumor activation of AMs, but restrains their antitumor activity to promote lung tumors via limiting RelA activation and/or activity, which could be through direct binding of p100 to RelA, sequestering RelA in the cytoplasm and restricting it from entering the nucleus to regulate gene expression. It is also required to maintain AMs during lung tumorigenesis. Of note, overactivation of RelA induces cell death, although it is often required for cell survival, including AM survival, in lung tumorigenesis (5, 49). Through p100 inhibition of RelB, NF-κB2 also limits the expansion and activity of pulmonary DCs for T cell suppression and lung tumor promotion. But cell-intrinsic NF-κB2 is also required for T cell activation and B cell maintenance for lung tumor suppression. These tumor-suppressive roles of lymphocyte NF-κB2 are consistent with the requirement of p52 generation from inducible p100 processing for B cell development and T cell activation during physiological conditions (18, 26, 50). Thus, NF-κB2 plays very important and complex roles in lung cancer (Figure 8).

These findings not only support dominant but complicated roles of immunity in host defense and tumor pathogenesis, but also provide a molecular and cellular basis to target myeloid NF-κB2 to restore antitumor immunity for lung cancer prevention and treatment. In this regard, myeloid NF-κB2 could be efficiently knocked down in cancer patients by NF-κB2 siRNAs conjugated with the Toll-like receptor 9 (TLR9) agonists CpG oligonucleotides (50). Clinically feasible mannose-conjugated nanoparticles can also be used to selectively deliver NF-κB2 siRNAs into AMs and TAMs, which highly express the mannose receptor CD206 (29, 51–54). NF-κB2–based immunotherapy can be used independently or combined with conventional chemoradiotherapies and innovative immunotherapies, particularly anti–PD-L1 antibodies, to treat lung cancer. Chemoradiotherapies induce PD-L1 expression on lung tumor cells and T cell tumor infiltration (14), while NF-κB2 knockout/knockdown in AMs and TAMs boosts T cells, providing a basis for their combination with each other and PD-L1 blockade therapy for lung cancer treatment. Myeloid RelA also promotes lung cancer and may be targeted for lung cancer treatment as well (5). However, targeting myeloid NF-κB2 should be a much better approach, because the lung cancer suppression induced by myeloid RelA deletion is not effective as that seen in NF-κB2–KO mice in the same urethane model (5). Given the antitumor role of lymphocyte NF-κB2, deletion of myeloid NF-κB2 alone will be more effective than global NF-κB2 deletion. It is worth considering and testing whether simultaneously targeting myeloid NF-κB2 and RelA, and perhaps also tumor RelA, shows better efficacy, since lung cancer suppression by myeloid RelA and NF-κB2 deletions involves different mechanisms, whereas intrinsic RelA promotes lung cancer as well (5, 12, 14). This is particularly important and interesting, given the failure of global NF-κB blockade in the clinic due to the complex roles and physiological importance of NF-κB, as well as the failure of all current therapies, including the combination of chemotherapy and PD-L1 blockade, in most cancer patients, including those with lung cancers.

In summary, the presented data indicate a cell type–dependent function for NF-κB2 in lung cancer. NF-κB2 drives myeloid AMs and DCs to promote and B and T lymphocytes to repress lung cancer, with a negligible or no role in nonimmune cells, including precancerous and tumor cells, and an overall protumor activity. These studies greatly increase our understanding of NF-κB and lung cancer, and importantly, suggest a feasible and effective NF-κB2–based immunotherapy for the deadliest cancer. Given the critical roles of TAMs in the pathogenesis and therapy resistance of many other tumors, these studies are highly relevant to the cancer field at large.

## Methods

### Sex as a biological variable.

Our study examined male and female animals, and similar results were obtained for both sexes.

### Animals and lung carcinogenesis.

All animals were maintained under pathogen-free conditions. NF-κB2–KO mice were originally obtained from Deborah Veis Novack (Washington University, St. Louis, Missouri, USA). NF-κB2–KO mice under a pure BALB/c background have been described previously (27). For lung carcinogenesis, 6- to 8-week-old mice were intraperitoneally (i.p.) injected with urethane (1 mg/g body weight, Sigma-Aldrich) once a week for 6 consecutive weeks (27). Mice were euthanized at 1 or 6 weeks after urethane treatment for examination of lung tumors and inflammation. Surface tumors in mouse lungs were counted blinded under a dissecting microscope and were measured by microcalipers. Some mice were euthanized 2 days after treatments with urethane (1 mg/g body weight, every other day for 6 total treatments) to examine macrophage apoptosis (Figure 6B).

### BM-chimeric mouse generation.

NF-κB2–KO and WT mice were irradiated with a single dose of 8.0 Gy. Eight hours later, the irradiated recipient mice were injected intravenously (i.v.) with 1.0 × 10^7^ BM cells from NF-κB2–KO or WT donor mice in 200 μL sterile PBS.

### In vivo BrdU labeling.

Mice were i.p. injected with 50 mg/kg BrdU (Sigma-Aldrich) 24 hours prior to euthanasia. Mouse lung tissue sections were stained with anti-BrdU (Sigma-Aldrich). More than 500 cells per mouse were counted in randomly selected tumor areas. The BrdU labeling index was calculated as the percentage of labeled cells per total cells counted. Mouse lung tissues with BrdU labeling were also used for flow cytometry analysis.

### Histology and immunohistochemistry analysis.

Lung and tumor tissues were excised, fixed in formalin, embedded in paraffin, and cut into 4-μm-thick sections. Sections were stained with H&E for histology, or subjected to sequential incubations with the indicated primary antibodies, biotinylated secondary antibodies, and streptavidin-horseradish peroxidase (HRP) for immunohistochemistry (IHC).

### In vitro tumor antigen–dependent T cell activation and tumor cell killing.

As previously described (5, 28), BMDCs from the indicated mice were pulsed with lysates of the mouse lung tumor cell line LLC stably expressing luciferase (LLC-Luc). Pulsed BMDCs were then cocultured with splenic CD3^+^ T cells from the indicated mice (1:5 ratio) in the presence of IL-2 (50 U/mL) for 4 days, followed by FACS analysis to detect IFN-γ^+^CD4^+^, granzyme B^+^CD4^+^, CD69^+^CD4^+^, IFN-γ^+^CD8^+^, granzyme B^+^CD8^+^, and CD69^+^CD8^+^ T cells. T cells isolated from BMDC coculture were further cocultured with LLC-Luc at the indicated ratio for 4 hours, followed by luciferase activity measure in the supernatant (indication of cell apoptosis, as luciferase can be released into the medium only after cell death). For macrophage-mediated tumor cell killing, BMDMs cultured in LLC-Luc TCM for 4 days were further cocultured with LLC-Luc at the indicated ratio for 24 hours, followed by luciferase activity measure in the supernatant.

### Flow cytometry analysis.

The cells were incubated with antibodies against cell surface antigens after blocking with anti-CD16/anti-CD32. The cells were then fixed with paraformaldehyde (2%), permeabilized with saponin (0.5%), and incubated with antibodies against intracellular antigens if needed. The cells from BrdU-labeled mice were stained with fluorochrome-conjugated BrdU antibody following cell surface protein staining, fixation and permeablization with BrdU Staining Buffer, and DNase I digestion. For IFN-γ staining, cells were treated with PMA (50 ng/mL), ionomycin (1 μM), brefeldin A (BFA, 3 μg/mL), and monensin (2 μM) for 4 hours before they were stained for flow cytometry analysis. Data were acquired and analyzed by Accuri C6 or LSRFortessa I (BD Biosciences) and FlowJo software (55).

### Bronchoalveolar lavage.

Upon euthanasia, mice lungs were lavaged with PBS as described previously (56). The recovered bronchoalveolar lavage fluids were centrifuged. Pelleted cells from bronchoalveolar lavage fluids were used for quantitative PCR (qPCR), immunofluorescence (IF), IHC, and/or FACS analysis.

### Cell lines and culture.

The mouse lung cancer cell line LLC-Luc and the human lung cancer cell lines H460 and H727 were obtained from Per H. Basse and Timothy F. Burns (University of Pittsburgh, Pittsburgh, Pennsylvania, USA) (14). These cells were cultured in RPMI 1640 supplemented with 1% or 10% FBS as indicated in the figures. The gene-expressing stable cell lines were generated using the retroviral vector pQCXIP. To avoid variations of different single-cell clones, bulk cells after puromycin selection and gene expression validation were used for all the assays.

### qPCR analysis.

The indicated tissues or cells were subjected to DNA or RNA extraction, RNA reverse transcription, and real-time PCR using TRIzol, reverse transcriptase, and Power SYBR Green PCR Master Mix (Thermo Fisher Scientific) according to the product manufacturer’s protocol.

### IF analysis.

Cells were fixed, permeabilized, and subsequently incubated with the indicated primary antibodies, followed by FITC- or TRITC-conjugated secondary antibodies (57, 58). Cells were also counterstained with DAPI for nuclear staining. Stained proteins and their subcellular localizations were detected using a Nikon Eclipse E800 (100×, 1.40 NA, oil objective) fluorescence microscope and analyzed by ImageJ software (NIH).

### Subcellular fractionation and immunoblotting assays.

For immunoblotting (IB), nuclear extracts were prepared by lysing pellets in nuclear buffer designed to dissolve insoluble proteins (20 mM Tris, pH 8.0, 150 mM NaCl, 1% [wt/vol] SDS, 1% [vol/vol] NP-40, and 10 mM iodoacetamide) after the cytoplasm was extracted with hypotonic buffer (20 mM HEPES, pH 8.0, 10 mM KCl, 1 mM MgCl_2_, 0.1% [vol/vol] Triton X-100, and 20% [vol/vol] glycerol) (59). The purity of the nuclear fractions was confirmed by checking Hsp90 (cytoplasm) or Sp1 (nuclear fraction). All the lysis buffers were supplemented with 1 mM PMSF and a protease inhibitor cocktail (Roche Molecular Biochemicals). The cell extracts were used for IB assays (60). Briefly, the cell extracts were resolved in polyacrylamide gels followed by electrotransfer onto nitrocellulose membranes. After blocking nonspecific protein binding with 5% dry milk, the membranes were sequentially incubated with appropriate primary and HRP-conjugated secondary antibodies and extensively washed with PBS with 0.1% Tween 20 (PBST) after each of the incubation steps. Specific immune complexes were detected by ECL as specified by the manufacturer (Western Lightning ECL Pro, Amersham).

### ChIP assays.

Cells were collected after formaldehyde treatment. The chromatin DNA was extracted, broken into fragments of 300–1000 bp in length by sonication, and immunoprecipitated with the indicated antibodies (61). DNA in the IP product was amplified by PCR.

### Colony formation assays.

Soft agar assays were performed as previously described (62–64). Briefly, cell suspensions in culture medium containing 0.6% SeaPlaque low-melting agarose were plated on the top of 1% agarose in culture medium. Colony growth was scored after 21 days of cell incubation. All the colony formation assays presented in this study were repeated in at least 3 independent experiments.

### Antibodies and primers.

Antibodies used for IF, IHC, ChIP, FACS, IB, and in vivo depletion assays, including the company names, catalog numbers, and dilutions, are listed in Supplemental Table 2. Primers for ChIP and qPCR are listed in Supplemental Table 3.

### Statistics.

Measurements were taken from distinct samples. Student’s *t* test (2 tailed, unpaired) was used to assess significance of differences between 2 groups. Ordinary 1-way ANOVA was used to assess significance of differences among groups of more than 2. Log-rank test was used to compare overall patient survival between high and low NF-κB2 expression groups. The survival analysis was justified with cancer stage, and demographic information, including sex, age, and smoking status of the patients with lung cancer, using Fisher’s exact test or χ^2^ test. All bars in figures represent mean ± SEM. *P* values less than 0.05 and 0.01 were considered statistically significant and highly statistically significant, respectively.

### Study approval.

We have complied with all relevant ethical regulations for animal testing and research. The animal experiments were performed in accordance with the US NIH *Guide for the Care and Use of Laboratory Animals* (National Academies Press, 2011). All animals were used according to protocols approved by the Institutional Animal Care and Use Committee (IACAUC) of the University of Pittsburgh and the University of Southern California.

### Data availability.

The TCGA lung cancer data we analyzed were obtained from https://portal.gdc.cancer.gov/projects (accessed 2017–2022). All data are included in the Supporting Data Values file. Any data that support the findings of this study are available from the corresponding authors upon reasonable request.

## Author contributions

FS designed, performed, and analyzed experimental assays. YX contributed to mouse clone maintenance and IHC staining. SDS provided advice and constructive feedback, and edited the manuscript. ZQ and GX conceived and designed the study, led and contributed to all aspects of the analysis, and wrote the manuscript.

## Supplementary Material

Supplemental data

Unedited blot and gel images

Supporting data values

## Version 1 02/22/2024

Electronic publication
