# Supplementary material for: Critical and distinct roles of cell type–specific NF-κB2 in lung cancer
Source: JCI Insight. 2024 Feb 22;9(4):e164188. doi: 10.1172/jci.insight.164188 (PMC10967404; doi:10.1172/jci.insight.164188)
Supplement: Supplemental data [file jciinsight-9-164188-s099.pdf]

Supplemental Table 1. Significance of difference between the tumoricidal activities of different T cells activated by different DCs (p values).

T: Tumor	T-WT, DC WT vs KO	T-KO, DC WT vs KO	DC-WT, T WT vs KO	DC-KO, T WT vs KO
100:1	3.54E-05	4.12E-06	4.67E-02	2.50E-04
10:1	8.01E-05	6.63E-05	7.23E-02	4.95E-04
1:1	1.68E-02	1.91E-02	6.23E-01	9.81E-01

Supplemental Table 2. Antibodies Used

Antibody	Clone	Catalog number	Company	Usage	Purpose
Anti-CD45 Alexa Fluor 700	30-F11, Rat IgG2b, κ	103128	Biolegend, San Diego, CA, USA	0.5 µl per sample	FACS
Anti-CD117 BV421	ACK2, Rat IgG2b, κ	135124	Biolegend, San Diego, CA, USA	5.0 µl per sample	FACS
Anti-NKp46 BV421	29A1.4, Rat IgG2a, κ	137612	Biolegend, San Diego, CA, USA	2.5 µl per sample	FACS
Anti-B220 BV510	RA3-6B2, Rat IgG2a, κ	103248	Biolegend, San Diego, CA, USA	2.5 µl per sample	FACS
Anti-Ly6G BV510	1A8, Rat IgG2a, κ	127633	Biolegend, San Diego, CA, USA	2.5 µl per sample	FACS
Anti-CD64 PE-Cy7	X54-5/7.1, Mouse IgG1, κ	139314	Biolegend, San Diego, CA, USA	2.5 µl per sample	FACS
Anti-CD69 PE-Cy5	H1.2F3, Armenian hamster IgG	104510	Biolegend, San Diego, CA, USA	1.25 µl per sample	FACS
Anti-FcεRIα PE-Cy7	MAR-1, Armenian Hamster IgG	134318	Biolegend, San Diego, CA, USA	2.5 µl per sample	FACS
Anti-BrdU FITC	BU20A, Mouse IgG1, κ	11-5071	Thermo Fisher Scientific, Waltham,MA, USA	5 µl per sample	FACS
Anti-MerTK Super Bright 436	DS5MMER, Rat IgG2a, κ	62-5751	Thermo Fisher Scientific, Waltham,MA, USA	5.0 µl per sample	FACS
Anti-MHC-II APC	M5/114.15.2, Rat IgG2b, κ	17-5321	Thermo Fisher Scientific, Waltham,MA, USA	0.15 µl per sample	FACS
Anti-CD11c APC-eFluor780	N418, Armenian hamster IgG	47-0114	Thermo Fisher Scientific, Waltham,MA, USA	2.5 µl per sample	FACS
Anti-CD4 APC-eFluor780	RM4-5, Rat IgG2a, κ	47-0042	Thermo Fisher Scientific, Waltham,MA, USA	0.625 µl per sample	FACS
Anti-CD11b PE	M1/70, Rat IgG2b, κ	12-0112	Thermo Fisher Scientific, Waltham,MA, USA	0.625 µl per sample	FACS
Anti-CD16/CD32	93, Rat IgG2a, λ	14-0161	Thermo Fisher Scientific, Waltham,MA, USA	1.0 µl per sample	FACS
Anti-CD3 PE	145-2C11, Armenian hamster IgG	12-0031	Thermo Fisher Scientific, Waltham,MA, USA	2.5 µl per sample	FACS
Anti-CD44 PE-Cy7	IM7, Rat IgG2b, κ	25-0441	Thermo Fisher Scientific, Waltham,MA, USA	0.625 µl per sample	FACS
Anti-CD4 PE-Cy7	RM4-5, Rat IgG2a, κ	25-0042	Thermo Fisher Scientific, Waltham,MA, USA	1.25 µl per sample	FACS
Anti-CD8 APC	53-6.7, Rat IgG2a, κ	17-0081	Thermo Fisher Scientific, Waltham,MA, USA	0.625 µl per sample	FACS
Anti-Granzyme B FITC	NGZB, Rat IgG2a, κ	11-8898	Thermo Fisher Scientific, Waltham,MA, USA	0.25 µl per sample	FACS
Anti-IFNγ FITC	XMG1.2, Rat IgG1, κ	11-7311	Thermo Fisher Scientific, Waltham,MA, USA	1.0 µl per sample	FACS
Anti-CD11c FITC	N418, Armenian hamster IgG	11-0114	Thermo Fisher Scientific, Waltham,MA, USA	0.5 µl per sample	FACS
Anti-Siglec-F PerCP-eFluor710	1RNM44N, Rat IgG2a, κ	46-1702	Thermo Fisher Scientific, Waltham,MA, USA	0.625 µl per sample	FACS
Anti-BrdU FITC	BU20A, Mouse IgG1, κ	11-5071	Thermo Fisher Scientific, Waltham,MA, USA	5 µL (1 µg) per sample	FACS
Ultra-LEAF™ Purified anti-mouse CD20 Antibody	SA271G2, Rat IgG2b, κ	93735	Biolegend, San Diego, CA, USA	200 µg/mouse (*iv*)	B cell depletion
Rat IgG1 κ Isotype Control, FITC	eBRG1	11-4301	Thermo Fisher Scientific, Waltham,MA, USA	1.0 µl per sample	FACS
Rat IgG2a, κ Isotype Ctrl, BV421	RTK2758	400549	Biolegend, San Diego, CA, USA	2.5 µl per sample	FACS
Rat IgG2a, κ Isotype Ctrl, BV510	RTK2758	400553	Biolegend, San Diego, CA, USA	2.5 µl per sample	FACS
Rat IgG2a κ Isotype Control, FITC	eBR2a	11-4321	Thermo Fisher Scientific, Waltham,MA, USA	0.25 µl per sample	FACS
Rat IgG2a κ Isotype Control, APC	eBR2a	17-4321	Thermo Fisher Scientific, Waltham,MA, USA	0.625 µl per sample	FACS
Rat IgG2a κ Isotype Control, APC-eFluor780	eBR2a	47-4321	Thermo Fisher Scientific, Waltham,MA, USA	0.625 µl per sample	FACS
Rat IgG2a κ Isotype Control, Super Bright 436	eBR2a	62-4321	Thermo Fisher Scientific, Waltham,MA, USA	5.0 µl per sample	FACS
Rat IgG2a κ Isotype Control,PerCP-eFluor710	eBR2a	46-4321	Thermo Fisher Scientific, Waltham,MA, USA	0.625 µl per sample	FACS
Rat IgG2b κ Isotype Control, Alexa Fluor® 700	RTK4530	400628	Biolegend, San Diego, CA, USA	0.5 µl per sample	FACS
Rat IgG2b, κ Isotype Control, APC	RTK4530	400611	Biolegend, San Diego, CA, USA	2.5 µl per sample	FACS
Rat IgG2b, κ Isotype Ctrl, BV421	RTK4530	400639	Biolegend, San Diego, CA, USA	5.0 µl per sample	FACS
Rat IgG2b κ Isotype Control, PE	eB149/10H5,	12-4031	Thermo Fisher Scientific, Waltham,MA, USA	0.625 µl per sample	FACS
Rat IgG2b κ Isotype Control, PE-Cyanine7,	eB149/10H5	25-4031	Thermo Fisher Scientific, Waltham,MA, USA	0.625 µl per sample	FACS
Armenian Hamster IgG Isotype Ctrl, PE/Cyanine5	HTK888	400909	Biolegend, San Diego, CA, USA	1.25 µl per sample	FACS
Armenian Hamster IgG Isotype Control, APC-eFluor 780,	eBio299Arm	47-4888	Thermo Fisher Scientific, Waltham,MA, USA	2.5 µl per sample	FACS
Armenian Hamster IgG Isotype Control, PE-Cyanine7	eBio299Arm	25-4888	Thermo Fisher Scientific, Waltham,MA, USA	2.5 µl per sample	FACS
Armenian Hamster IgG Isotype Control, PE	eBio299Arm	12-4888	Thermo Fisher Scientific, Waltham,MA, USA	2.5 µl per sample	FACS
Mouse IgG1, κ Isotype Ctrl, PE-Cy7	MOPC-21	400125	Biolegend, San Diego, CA, USA	2.5 µl per sample	FACS
Mouse IgG1 κ Isotype Control, FITC	P3.6.2.8.1	11-4714	Thermo Fisher Scientific, Waltham,MA, USA	2 µl per sample	FACS
Anti-Arginase	N-20, goat polyclonal IgG	sc-18351	Santa Cruz Biotechnology, Dallas, TX,USA	1:500	IF

Antibody	Clone	Catalog number	Company	Usage	Purpose
Anti-iNOS	Rabbit polyclonal IgG	ab15323	Abcam, Cambridge, MA, USA	1:100	IF
Anti-RelA	D14E12, Rabbit IgG	8242	Cell Signaling Technology, Danvers,MA, USA	1:800 (IF); 1:100 (ChIP)	IF/ChIP
anti-HIF-1 alpha	H1alpha67, mouse IgG2b	NB100-105	Novus Biologicals, Littleton, CO, USA	1:100	ChIP
anti-HIF-2 alpha/EPAS1	Rabbit polyclonal	NB100-122	Novus Biologicals, Littleton, CO, USA	1:100	ChIP
Anti-F4/80	Cl:A3-1, Rat IgG2b	MCA497G	Bio-Rad, Hercules, CA, USA	1:200	IF
Anti-CD68	FA-11, Rat IgG2a, κ	14-0681	Thermo Fisher Scientific, Waltham,MA, USA	1:200	IF
Anti-BrdU	BU-33, Mouse monoclonal	B2531	Sigma-Aldrich, St. Louis,MO, USA	1:500	IHC
Anti-CD34	C-18, goat polyclonal IgG	sc-7045	Santa Cruz Biotechnology, Dallas, TX,USA	1:500	IHC
Anti-Cleaved Caspase 3 (Asp175)	Rabbit polyclonal	9661	Cell Signaling Technology, Danvers,MA, USA	1:200	IHC
Anti-RelB	Rabbit polyclonal	4954	Cell Signaling Technology, Danvers,MA, USA	1:1000	IB
NF-κB1 p105/p50 Rabbit mAb	D4P4D, Rabbit monoclonal	13586	Cell Signaling Technology, Danvers,MA, USA	1:1000	IB
Anti-Sp1 (PEP 2)	Rabbit polyclonal IgG	sc-59	Santa Cruz Biotechnology, Dallas, TX,USA	1:200	IB
Anti-Hsp90	F-8, Mouse monoclonal IgG2a	sc-13119	Santa Cruz Biotechnology, Dallas, TX,USA	1:1000	IB
Anti-mouse IgG Biotinylated		BMK-2202	Dako, Carpinteria, CA, USA	1:200	IHC
Rabbit anti-Goat IgG Biotinylated		sc-2774	Santa Cruz Biotechnology, Dallas, TX,USA	1:200	IHC
Goat anti-Rabbit IgG Biotinylated		E0432	Dako, Carpinteria, CA, USA	1:200	IHC
Donkey anti-Goat TRITC		A16004	Thermo Fisher Scientific, Waltham,MA, USA	1:200	IF
Donkey anti-Rat FITC		DKXRT-003-DFITC	ImmunoReagents Inc., Raleigh, NC, USA	1:200	IF
Donkey anti-Rabbit IgG-R		sc-2095	Santa Cruz Biotechnology, Dallas, TX,USA	1:200	IF
Donkey anti-Goat IgG-FITC		sc-2024	Santa Cruz Biotechnology, Dallas, TX,USA	1:200	IF
Donkey anti-Rabbit IgG-FITC		sc-2090	Santa Cruz Biotechnology, Dallas, TX,USA	1:200	IF
Goat anti-Mouse HRP		sc-2055	Santa Cruz Biotechnology, Dallas, TX,USA	1:5000	IB
Donkey anti-Goat HRP		sc-2020	Santa Cruz Biotechnology, Dallas, TX,USA	1:5000	IB
Goat anti-Rabbit IgG-HRP		sc-2054	Santa Cruz Biotechnology, Dallas, TX,USA	1:5000	IB

Supplemental Table 3. Primers Used

Gene	Species	Accession number	Forward (5' to 3')	Reverse (5' to 3')	Usage
18S RNA	mouse	NR_003278.3	AGGAATTGACGGAAGGGCAC	GGACATCTAAGGGCATCACA	RT-PCR
β-actin	mouse	NM_007393.3	ACCCGCGAGCACAGCTTCTTTG	CTTTGCACATGCCGGAGCCGTTG	RT-PCR
Arginase1	mouse	NM_007482.3	AATCTGCATGGGCAACCTGT	GTCTACGTCTCGCAAGCCAA	RT-PCR
iNOS	mouse	NM_010927.3	ACGAGACGGATAGGCAGAGA	GCACATGCAAGGAAGGGAAC	RT-PCR
Vegfa	mouse	NM_009505.4	GCACTGGACCCTGGCTTTAC	GTCTCAATCGGACGGCAGTA	RT-PCR
IL1b	mouse	NM_008361.3	CCTCGTGCTGTCGGACCCAT	GGACAGGTATAGATTCTTTCCTTTGAGGC	RT-PCR
IL12b	mouse	NM_001303244.1	CATCTCCTGGTTTGCCATCGTTT	GGGAGTCCAGTCCACCTCTACAACA	RT-PCR
Tnf	mouse	NM_013693.3	GATGAGAAGTTCCCAAATGGC	ACTTGGTGGTTTGCTACGACG	RT-PCR
HIF1a	mouse	NM_001313919.1	TGGACTTGTCTCTTTCTCCGC	TTCGACGTTCAGAACTCATCCT	RT-PCR
HIF2a	mouse	NM_010137.3	ACTTGGACGCTCTGCCTATG	TTGCGGGGGTTGTAGATGAC	RT-PCR
Arginase1	mouse	NC_000076.7	GTGGCTTTACACAGGGACCG	CCGATGACCCGAGTTTGACC	ChIP PCR, HRE1
Arginase1	mouse	NC_000076.7	TAAATCTCTGCCTAGCCCGC	GCCTAAGGCATCCTGCTATC	ChIP PCR, HRE2
iNOS	mouse	NC_000077.7	TCATGAATGAGCTAACTTGC	CAGCAGCCATCAGGTATTTA	ChIP PCR, kB site
iNOS	mouse	NC_000077.7	GCTGCAAGCCAGGGTATGTG	TCTCTTCCTGGTCGCCCGTC	ChIP PCR, HIF site
