# Supplementary material for: Critical and distinct roles of cell type–specific NF-κB2 in lung cancer
Source: JCI Insight. 2024 Feb 22;9(4):e164188. doi: 10.1172/jci.insight.164188 (PMC10967404; doi:10.1172/jci.insight.164188)

Full unedited gel for Figure 2A

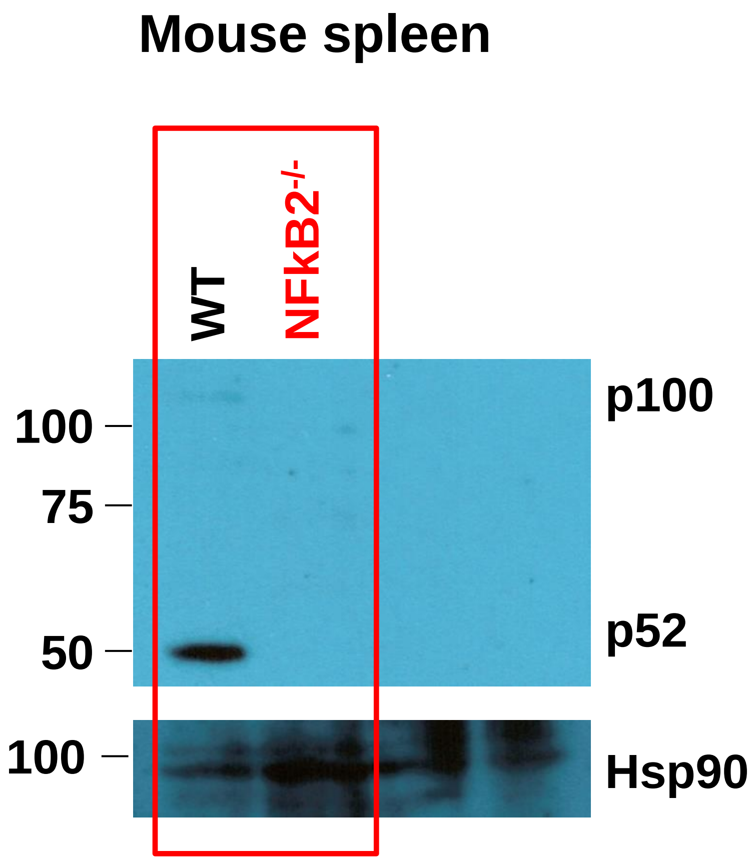

Full unedited gel for Figure 2B

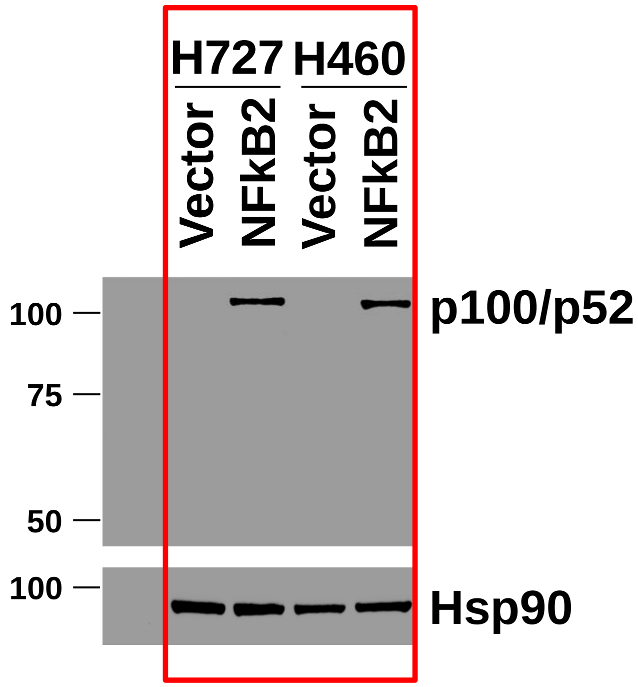

Full unedited gel for Figure 5F

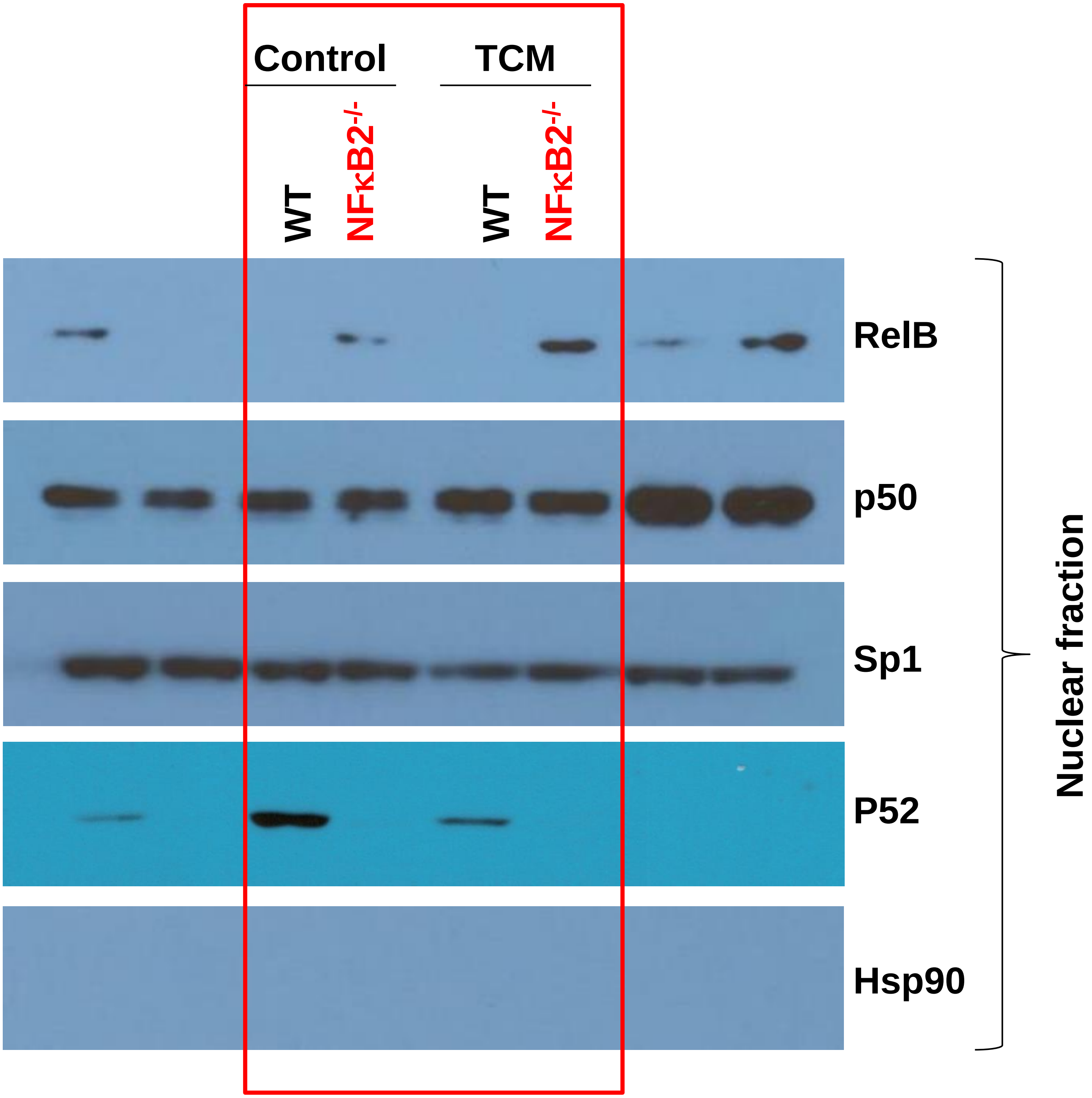

Supplement: Unedited blot and gel images [file jciinsight-9-164188-s100.pdf]
